# Integrated investigation of the clinical implications and targeted landscape for RNA methylation modifications in hepatocellular carcinoma

**DOI:** 10.1186/s40001-023-01016-7

**Published:** 2023-01-27

**Authors:** Jianping Zhang, Jie Gao, Mingchao Hu, Shiyu Xu, Chun Cheng, Wenjie Zheng, Jie Zhang

**Affiliations:** 1 Research Center of Clinical Medicine, Affiliated Hospital of Nantong University, Medical School of Nantong University, 20 Xisi Road, Nantong, 226001 Jiangsu China; 2 Department of Oncology, Yancheng First Hospital, Affiliated Hospital of Nanjing University Medical School, Yancheng, 224006 Jiangsu China; 3 Department of Oncology, Affiliated Hospital of Nantong University, Medical School of Nantong University, Nantong, 226001 China

**Keywords:** RNA methylation, Writer, Hepatocellular carcinoma, Tumor microenvironment, RM_Score, Drug sensitivity, Immunotherapy

## Abstract

**Background:**

RNA methylation (RM) is a crucial post-translational modification (PTM) that directs epigenetic regulation. It mostly consists of N^1^-methyladenosine (m^1^A), 5-methylcytosine (m^5^C), N^3^-methylcytidine (m^3^C), N^6^-methyladenosine (m^6^A), and 2′-O-methylation (Nm). The “writers” mainly act as intermediaries between these modifications and associated biological processes. However, little is known about the interactions and potential functions of these RM writers in hepatocellular carcinoma (HCC).

**Methods:**

The expression properties and genetic alterations of 38 RM writers were assessed in HCC samples from five bioinformatic datasets. Two patterns associated with RM writers were identified using consensus clustering. Then, utilizing differentially expressed genes (DEGs) from different RM subtypes, we built a risk model called RM_Score. Additionally, we investigated the correlation of RM_Score with clinical characteristics, tumor microenvironment (TME) infiltration, molecular subtypes, therapeutic response, immunotherapy effectiveness, and competing endogenous RNA (ceRNA) network.

**Results:**

RM writers were correlated with TME cell infiltration and prognosis. Cluster_1/2 and gene.cluster_A/B were shown to be capable of distinguishing the HCC patients with poor prognosis after consensus and unsupervised clustering of RNA methylation writers. Additionally, we constructed RNA modification pattern-specific risk model and subdivided the cases into RM_Score high and RM_Score low subgroups. In individual cohorts or merged datasets, the high RM_Score was related to a worse overall survival of HCC patients. RM_Score also exhibited correlations with immune and proliferation related pathways. In response to anti-cancer treatments, the RM_Score had a negative correlation (drug sensitive) with drugs that focused on the MAPK/ERK and metabolism signaling, and a positive correlation (drug resistant) with compounds targeting RKT and PI3K/mTOR signaling pathway. Notably, the RM_Score was connected to the therapeutic effectiveness of PD-L1 blockage, implying that RM writers may be the target of immunotherapy to optimize clinical outcomes. Additionally, a ceRNA network was generated including 2 lncRNAs, 4 miRNAs, and 7 mRNAs that was connected to RM writers.

**Conclusions:**

We thoroughly investigated the potential functions of RNA methylation writers and established an RM_patterns-based risk model for HCC patients. This study emphasized the critical functions of RM modification in TME infiltration, targeted therapy, and immunotherapy, providing potential targets for HCC.

**Supplementary Information:**

The online version contains supplementary material available at 10.1186/s40001-023-01016-7.

## Introduction

Hepatocellular carcinoma (HCC), accounting for approximately 90% of liver cancer, is the major histologic type of liver cancer. Annually, more than a million individuals are newly diagnosed with liver cancer, raising concerns on the world’s health [1]. As an inflammation-associated malignancy, HCC is frequently caused by a number of risk factors, including drinking, nonalcoholic steatohepatitis (NASH), and hepatitis B virus (HBV)/hepatitis C virus (HCV) infection [2]. In contrast to other solid cancers, HCC is commonly diagnosed at an advanced stage, until which it is too late for transplantation, surgical resection, or local ablation. By decades, long-term improvements have been made in the management of advanced HCC. Sorafenib was first approved as an effective systemic option for advanced HCC. More recently, other targeted drugs such as lenvatinib, regorafenib, cabozantinib, nivolumab, and ramucirumab have also displayed clinical efficacy [3]. However, heterogeneity revealed by multiple omics poses a significant challenge for precision medicine and increases survival of HCC patients. Understanding the probable mechanisms of hepatocarcinogenesis, as well as searching for robust biomarkers and targets, will thus tremendously benefit HCC treatment.

Benefiting from high-throughput sequencing and large-scale profiling, post-translational modification (PTM) has been uncovered in a variety of RNAs, such as messenger RNA (mRNA), transfer RNA (tRNA), ribosomal RNA (rRNA) long noncoding RNA (lncRNA), and enhancer RNAs (eRNAs). RNA methylation (RM), including includes N^1^-methyladenosine (m^1^A), 5-methylcytosine (m^5^C), N3-methylcytidine (m^3^C), N6-methyladenosine (m^6^A), and 2′-O-methylation (Nm), is described as a crucial PTM in governing RNA maturation, splicing, stability, and translation. Among all these RNA methylation modifications, N6-methylation of adenosines is the most common and abundant RNA methylation modification that occurs at stop codons and within 3′ UTRs. The m^1^A mutation is likewise found at the first position of the adenine base. Recently, m^1^A is also reported enriched at translation start sites (5′ UTRs). Nm, which is found on the 2′ hydroxyl ribose moiety of ribonucleosides, has been found in all major eukaryotic RNA [4]. In contrast, m^5^C and m^3^C, two isomeric cytidine methylation modifications, present a methyl group on the nucleobase of cytidines. The newly identified RNA methylation type known as m^3^C differs from other well-known modification patterns. It was first discovered in rRNA and tRNA, in which it took part in modeling RNA structure and protein–RNA interactions [5, 6]. However, the m^3^C modification in mRNA of mice and human was also recently discovered [7].

Specific “writers” (methyltransferase), “readers” (binding proteins), and “erasers” (demethylases), in that order, can deposit, recognize, and remove labeled RNA methylation modifications, respectively. These enzymes have been connected to immune responses, cell differentiation, DNA replication, DNA repair, and other common biological processes. However, a growing number of studies have shown the critical functions of dysregulated modification modulators, particularly “writers”, in modulating the malignant phenotypes of various cancer types. For HCC, the oncogenic roles of m^6^A writers have drawn more attentions. Through the HuR-ETS1-p21/p27 axis, WTAP-induced m^6^A alteration enhanced the progression of HCC [8]. METTL3-mediated m^6^A modification contributed to sorafenib resistance of HCC cells in the hypoxic tumor microenvironment [9]. METTL14, on the other hand, impeded the invasion of HCC cells by suppressing EGFR/PI3K/AKT signaling [10]. Given the biological features of m^6^A writers, these inhibitors are promising drug candidates for tumor therapy, including FB23-2, R-2HG, BTYNB, and CS1 [11]. By recruiting H19 lncRNA to methylate, the typical m^5^C methylase NSUN2 accelerated carcinogenesis, cell migration, and invasion of HCC cells. 2′-O-methylation writer Fibrillarin (FBL) was correlated with advanced TNM stage and poor survival of HCC cases [12]. According to the most recent study, TRMT6/TRMT61A-mediated m^1^A methylation enhanced liver tumorigenesis and CSC development via activating Hedgehog and PPAR signaling [13]. More intriguingly, despite the fact that m^3^C has been specifically identified in the HBV-infected HCC cell line Huh7, the relationship between m^3^C writers and the evolution of HCC has not been fully understood [14]. It has been postulated that this methylation modification may play an important role in liver cancer brought on by viral infection.

Given the aforementioned, RNA methylation modification authors may be essential for the development of HCC. Though recent studies highlighted the significance of RNA methylation modification in tumorigenesis, the comprehensive analyses of the RNA methylation writers for HCC remain limited [15, 16]. In the current study, we investigated thoroughly the writers’ expression and mutation levels of 5 RNA methylation types (m^1^A, m^5^C, m^3^C, m^6^A, and Nm) in 5 HCC cohorts. Additionally, utilizing various clustering algorithms, we constructed an RNA methylation model to forecast tumor microenvironment (TME) alterations, immunotherapy response, and survival status. As a result, an integrated investigation of the patterns of RNA methylation may uncover the interaction regulatory mechanisms and provides potential HCC targets.

## Methods

### Data collection and processing

**Fig. 1:**
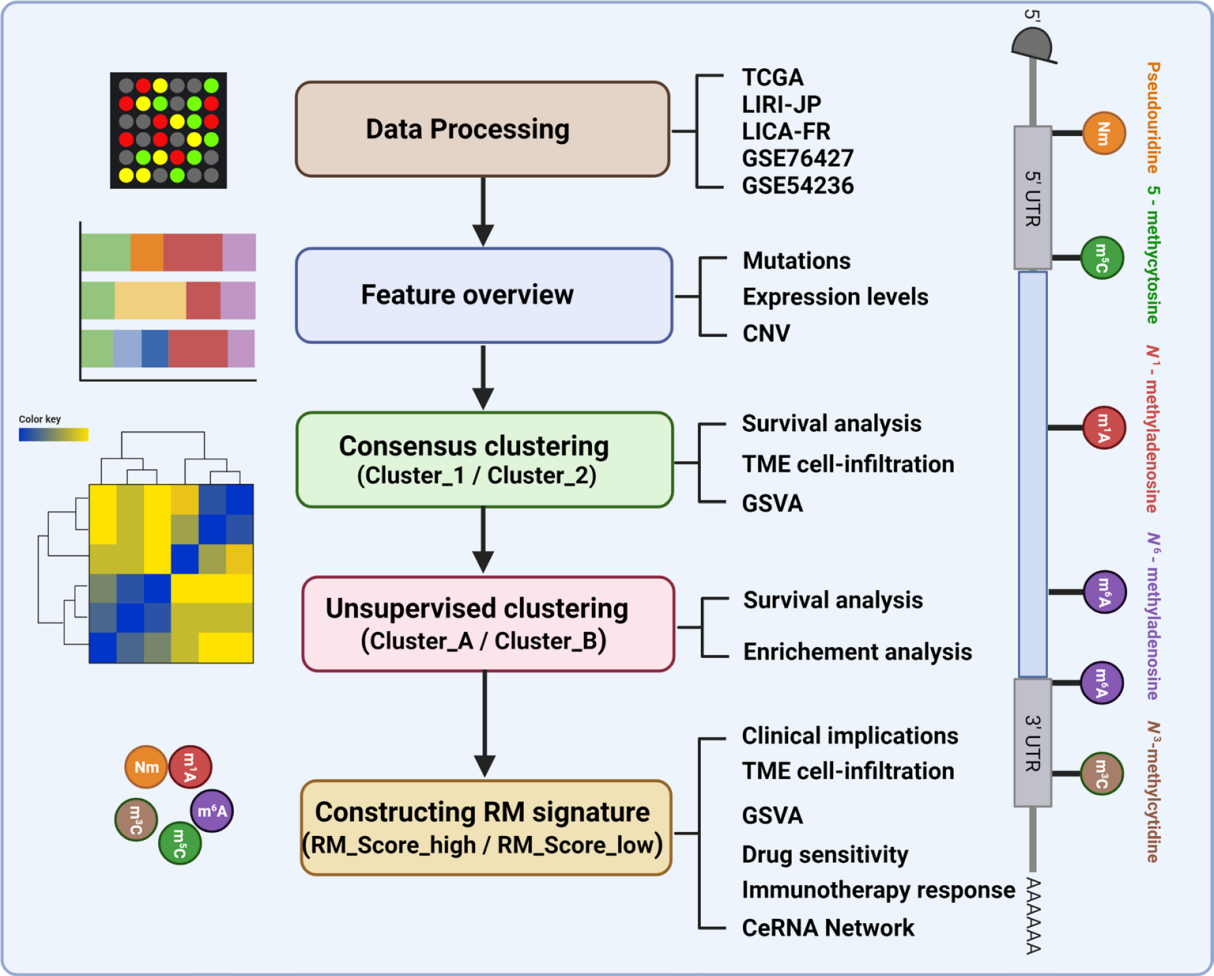
Flowchart of this study. The expression and mutation features of RNA methylated modification writers were investigated in 5 HCC datasets. Following clustering algorithms, a RNA methylation-based model was constructed to forecast tumor microenvironment alterations, immunotherapy response, targeted drug sensitivity, and survival status of HCC individuals

The study’s flowchart was depicted in Fig. 1 for reference. Gene expression levels and clinical information were downloaded from Gene-Expression Omnibus (GEO), the Cancer Genome Atlas (TCGA) database and ICGC database. mRNA expression, miRNA expression, lncRNA expression, somatic mutation, SCNAs were retrieved from TCGA database (https://portal.gdc.cancer.gov/). 2 GEO liver cancer cohorts (GSE76427 and GSE54236) [17, 18], 2 ICGC cohorts (LIRI-JP and LICA-FR), and TCGA-LIHC cohort were included for combined analysis (Additional file 2: Table S1). The log transformed FPKM values from TCGA, LIRI-JP, and LICA-FR were integrated with the gene array data (GSE76427 and GSE54236). Batch effects were corrected by using “ComBat” algorithm of svaPackage. IMvigor210 cohort, including 348 metastatic urinary tract transitional cell carcinoma cases administrated with atezolizumab, was included to evaluate the predictive value of RM_Score in predicting immunotherapy [19, 20]. The genomic, transcriptomic, and clinical data were obtained from http://research-pub.gene.com/IMvigor210CoreBiologies.

### Mutation status and copy number variations of the RM writers

A total of 38 RM writers were involved in this analysis (Additional file 2: Table S2), including m^6^A writers (METTL3, METTL14, METTL16, ZCCHC4, WTAP, RBM15, RBM15B, ZC3H13, KIAA1429), m^5^C writers (NOP2, NSUN2, NSUN3, NSUN4, NSUN5, NSUN6, NSUN7, TRDMT1), Nm writers (CMTR1, CMTR2, FBL, TRMT13, TRMT44, TARBP1, HENMT1, FTSJ1, FTSJ3, MRM1, FTSJ2, RNMTL1), m^1^A writers (TRMT6, TRMT61A, TRMT61B, TRMT10C, RRP8), and m^3^C writers (METTL2A, METTL2B, METTL6, METTL8). The “maftools” package was utilized to demonstrate the mutation levels of the RNA methylation writers in pan-cancers of TCGA dataset. The patients were split into groups with and without mutations, which were further compared for their overall survival. Copy number variations was calculated to evaluate the expression levels and copy number.

### Clustering expression pattern of RNA methylation “writers”

Following excluding genes missed in any of the datasets (NSNU7 missed in LIRI-JP, RBM15B and MRM1 missed in LICA-FR), a total of 35 writers covering the 5 types of RNA methylated modification were included for further analyses, including 11 Nm writers (CMTR1, CMTR2, FBL, TRMT13, TRMT44, TARBP1, HENMT1, FTSJ1, FTSJ3, FTSJ2, RNMTL1), 8 m^6^A modification enzymes (METTL3, METTL14, METTL16, ZCCHC4, WTAP, RBM15, ZC3H13, KIAA1429), 7 m^5^C writers (NOP2, NSUN2, NSUN3, NSUN4, NSUN5, NSUN6, TRDMT1), 4 m^3^C writers (METTL2A, METTL2B, METTL6, METTL8), and 5 m^1^A writers (TRMT6, TRMT61A, TRMT61B, TRMT10C, RRP8). Consensus clustering algorithm was performed to cluster analysis of 35 RM writers in combined liver cancer cohorts by performing “ConsensusClusterPlus” package with 100 repetitions.

### Gene set variation analysis and TME analysis

By using the "GSVA" R package, gene set variation analysis (GSVA) was performed to investigate the impact of RNA modification clusters on biological processes and pathways [21]. “c2.cp.kegg.v7.1.symbols.gmt” and “h.all.v7.4.entrez.gmt.txt” for GSVA analysis was downloaded from the MSigDB database (https://www.gsea-msigdb.org/gsea/index.jsp). 35 RM genes were functionally annotated using the clusterProfiler R Package. he levels of infiltration of 28 different types of immune cells were measured using single sample gene set enrichment analysis (ssGSEA) in the "GSVA" R package. By analyzing 547 immune-cell signature genes, CIBERSORT (https://cibersort.stanford.edu/) was used to determine the relative abundance of 22 different immune cell types.

### Construction of the RM_Score model

Using the Limma R package, DEGs associated to RNA methylation phenotype were identified between Cluster 1 and Cluster 2. Then we performed unsupervised clustering to obtain gene.cluster_A and gene.cluster_B. The prognostic value of DEGs was further determined using a univariate Cox regression model. Then, to establish a scoring system, DEGs related to survival were retrieved. RM_Score of each patient was calculated as follows: RMScore = $\sum {PC}_{1}+\sum {PC}_{2}$. RMScore is equal to PC 1 plus PC 2. The optimal cut-off value for the RM_Score, which separated the cases into high RM_Score group and low RM_Score group, was defined using the surv_cutpoint function in the survminer R package.

### Association analysis of RM_Score and drug sensitivity

The transcription profiles for about 1000 cancer cell lines, drug response measurements as AUC for antitumor drugs in cancer cell lines, and targets/pathways of drugs are downloaded from Genomics of Drug Sensitivity in Cancer (GDSC, http://www.cancerrxgene.org/downloads) [64]. We performed Spearman correlation analysis to calculate the correlation between drug sensitivity and RM_Score. |Rs|> 0.3 and FDR < 0.05 (Benjamini and Hochberg adjusted) were considered as significant correlation.

### Construction of competing endogenous RNA (ceRNA) network

miRNA–mRNA interactions were retrieved from the miRTarBase(http://mirtarbase.mbc.nctu.edu.tw/php/index.php), TargetScan (http://www.targetscan.org/vert_72/) and miRDB (http://mirdb.org/). miRNA–mRNA interactions overlapped in the three databases were utilized for further analysis. lncRNA–miRNA interactions were predicted by TargetScan. Cytoscape were performed to generate mRNA–miRNA–lncRNA network.

### Statistics

The data were analyzed using R (version 3.6.2) and R Bioconductor packages. Receiver operating characteristic (ROC) curve, Kaplan–Meier method and univariate/multivariate Cox regression model were used to verify the validity of the model. RCircos package was used to present the distribution of the RM writers in the chromosome. *p* value less than 0.05 was considered as statistically significant.

## Results

### Genetic alterations of five types of RM writers in HCC

**Fig. 2:**
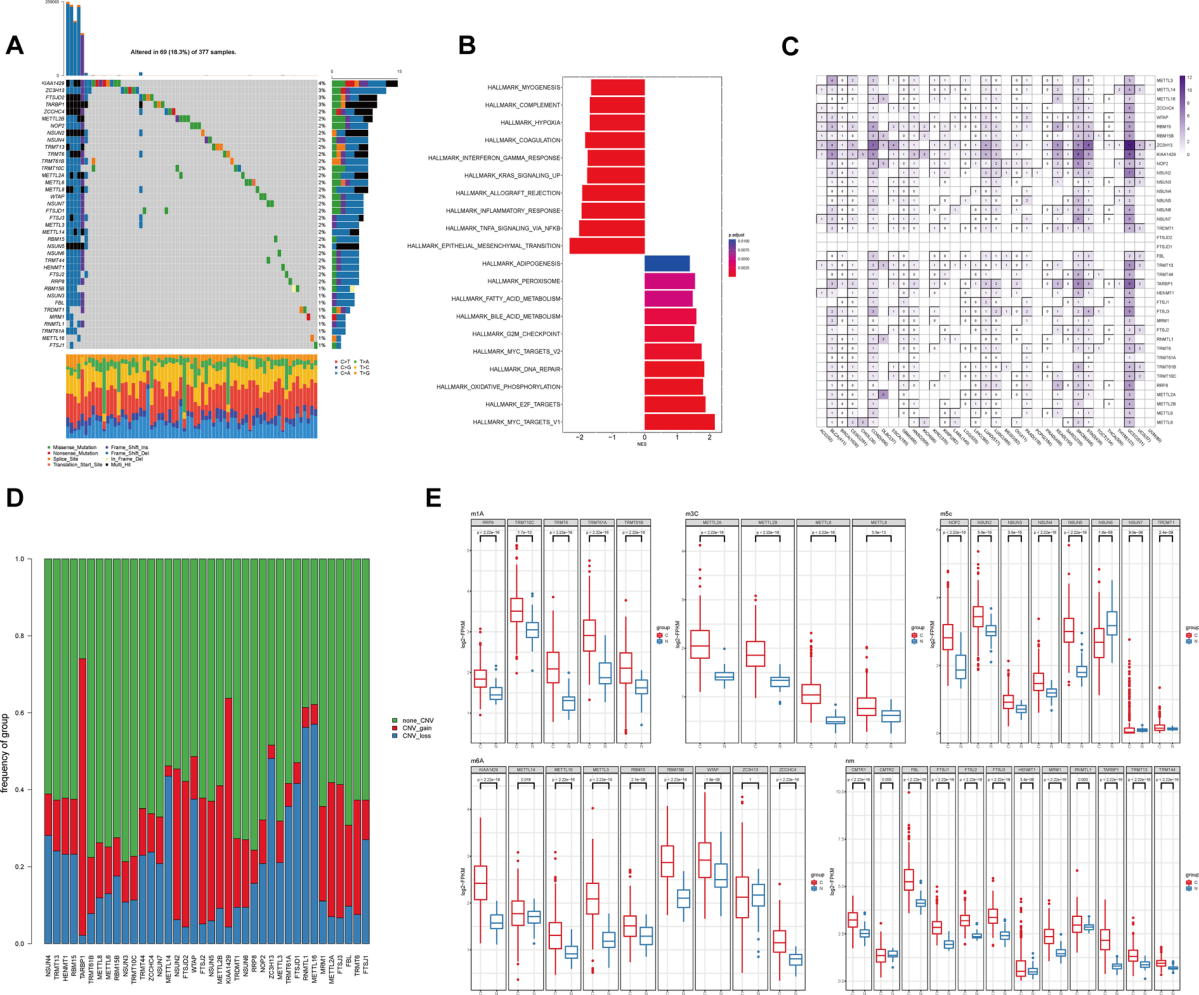
Comprehensive landscape of RNA methylated modification writers in liver cancer. **A** The mutation frequency of RNA modification writers in liver cancer patients from the TCGA cohort. **B** GSEA elucidating the samples with or without writer mutation. **C** The mutation landscape of the RNA modification writers among pan-cancers in TCGA cohort. **D** Different CNV types of RNA modification writers in the TCGA-LIHC cohort. **E** The expression of RNA modification writers in paired normal and cancer tissues in TCGA-LIHC cohort

38 “writers” of the five most prevalent RNA methylation modifications were analyzed in this study, including 5 m^1^A writers, 8 m^5^C writers, 9 m^6^A writers, 4 m^3^C writers, and 12 Nm writers. 69 of the 377 liver cancer samples tested positive for the mutations of writers mentioned above. Among them, KIAA1429 displayed relatively higher mutation frequency (Fig. 2A). Despite the fact that there were no appreciable differences in survival between HCC patients with or without mutations (Additional file 1: Fig. S1A, B), the poor prognosis of HCC patients was substantially associated with mutations of the 20 RNA methylated modification writers (FTSJ2, FTSJ3, FTSJD1, HENMT1, KIAA1429, METTL2A, METTL2B, METTL8, METTL14, NSUN2, NSUN3, NSUN4, NSUN5, NSUN6, NSUN7, RBM15, TRDMT1, TRMT6, TRMT44, and TRMT61B; Additional file 1: Fig. S1C, *P* < 0.05). It was speculated that the writers' genetic alterations may have contributed to the development of HCC.

To assess the differences between the individuals with and without mutation, Gene Set Variation Analysis (GSVA) was conducted using hallmark gene sets. As shown in Fig. 2B, the mutant group exhibited increased expression of cancer-related signals such as MYC targets, E2F targets, DNA repair, oxidative phosphorylation, and G2M checkpoint pathways. In contrast, TNFα signaling, inflammatory response, and interferon-γ response were downregulated. Next, we examined somatic copy number alterations of the RM writers. Of them, TARBP1, KIAA1429, MTEEL3, RBM15, RBM15B, and TARBP1 had extensive somatic mutation among pan-cancers (Fig. 2C). For HCC patients, the highest frequency of copy number variation (CNV) gain was observed in TARBP1 and KIAA1429, while the highest frequency of CNV loss was identified in RNMTL1 and METTL16 (Fig. 2D). Apart from the genetic variations, the majority of the writers had considerably higher levels of mRNA expression in HCC samples compared to that in normal tissues. In contrast, m^5^C writer NSUN6 displayed downregulated expression levels in HCC tissues (Fig. 2E). In order to comprehend more about the discrepancy between CNV and mRNA expression, we examined the subgroups of CNV levels, such as normal tissues, CNV gain, CNV loss, and/or non-significant CNV alterations (Additional file 1: Fig. S2). Indeed, patients with CNV gain showed frequently higher mRNA expression than those with CNV loss. However, ZC3H13, NSUN6, NSUN7, and RNMTL1 showed significant downregulation or non-significant alteration in CNV loss group compared to normal tissues. Thus, it suggested that CNV could partially modulate mRNA expression of the writers in HCC, indicating a high heterogeneity of genetic landscape and RM writer expression in HCC.

### Distinct patterns of RM writers associated with cancer hallmarks and immune infiltration

**Fig. 3:**
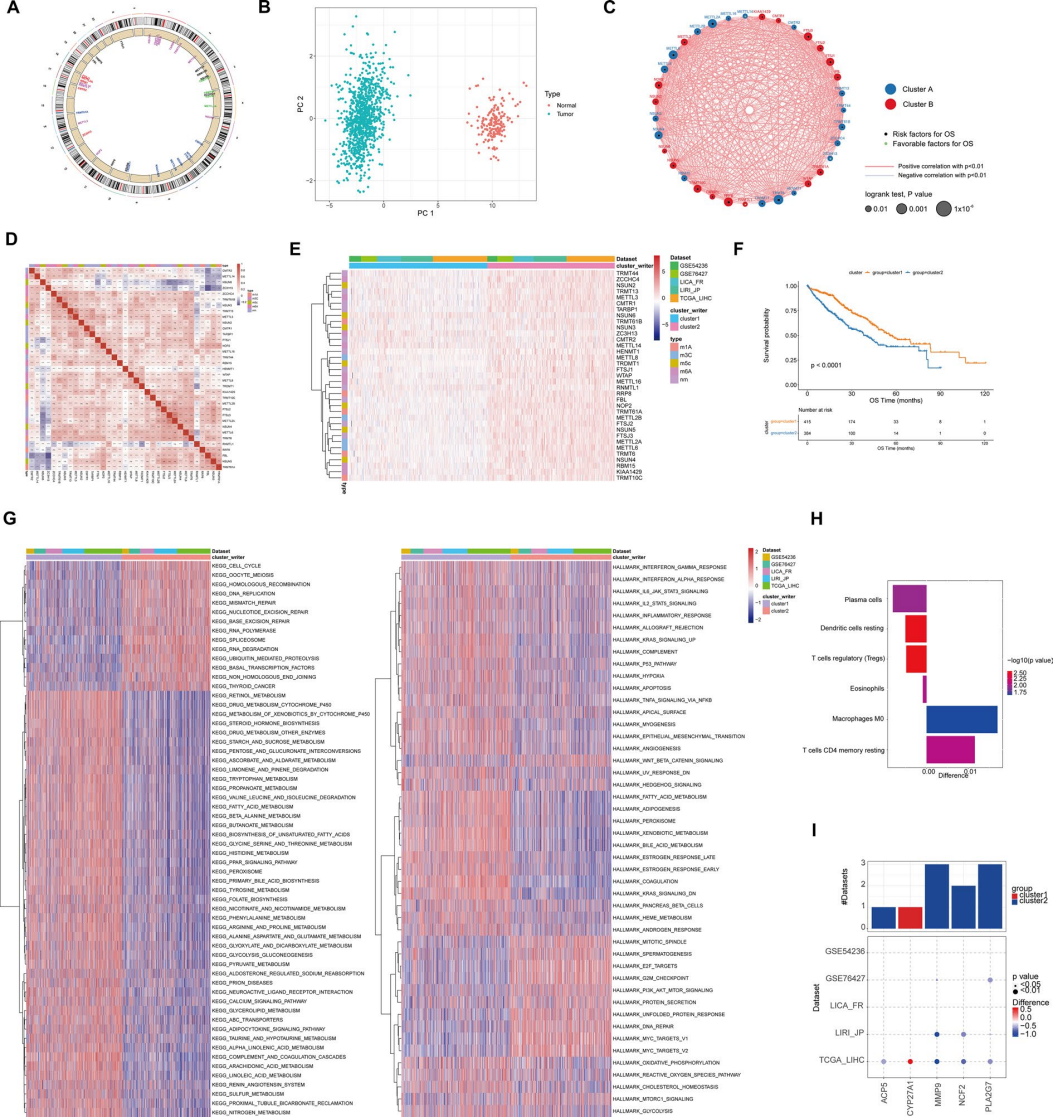
Cancer hallmarks and immune infiltration of the RNA methylation writers. **A** The genomic distribution of the RNA methylation writers. **B** PCA of the RNA methylation writers for distinguishing tumor and normal patients in TCGA-LIHC cohort. **C** The regulatory network of the RNA methylation writers based on the consensus analysis. **D** Spearman correlation analysis showed positive or negative correlation among RNA methylation writers in HCC. **E** Consensus clustering of RNA methylation writers in 5 cohorts. **F** Kaplan–Meier curves demonstrating the overall survival of Cluster_1 and Cluster_2. **G** GSVA enrichment analysis shows the correlation of the two clusters with biological pathways and cancer hallmarks. **H** The difference of immune cell infiltration in TME between Cluster_1 and Cluster_2. **I** The expression difference M0 macrophage and CD4 T cell between Cluster_1 and Cluster_2

As shown in Fig. 3A, the “writers” of the RNA methylation modifications exhibited distinct gene distributions. To comprehensively understand the expression pattern of the “writers” engaged in carcinogenesis, we selected five HCC datasets (TCGA, LIRI-JP, LICA-FR, GSE76427, and GSE54236) containing 955 HCC samples and 179 normal samples. Following excluding genes missed in any of the datasets, 5 writers covering 5 RM categories were recruited for further analysis. According to PCA analysis, these writers were able to effectively discriminate tumor samples and normal samples (Fig. 3B). Univariate Cox regression showed that 27 of 35 RM writers were substantially correlated with prognosis of HCC patients (Additional file 1: Fig. S3A). Additionally, consensus clustering showed that these writers had potentially negative or positive correlations (Fig. 3C).

The spearman correlation analysis found that the majority of these writers had favorable correlations. However, NSUN6 and ZC3H13 had weak or negative associations with other writers (Fig. 3D). It was hypothesized that the writers of the various RNA methylation modification patterns might interact with one another. After consensus clustering, 955 patients from the pooled datasets were stratified into Cluster_1 and Cluster_2 (Fig. 3E; Additional file 2: Table S3). Remarkably, patients at Cluster 1 had better prognosis than that of Cluster 2 modification pattern (Fig. 3F, *p* < 0.0001). Subsequently, we conducted GSVA enrichment analysis to explore the biological implications of these different RM patterns (Additional file 2: Table S4, Table S5). According to the top 60 enriched pathways from KEGG analysis, DEGs of the two Clusters were mainly enriched in metabolism and pathways associated with proliferation, spliceosome and RNA modification. In the Hallmark analysis, DEGs were substantially associated with a number of immunological and oncogenic pathways, including Kras signaling, PI3K/AKT/mTOR signaling, IL2/IL6 signaling, interferon α/γ response, and TNFA signaling (Fig. 3G), suggesting the potential role of “writers” in immune and TME. Actually, there may be a specific association between TME cell infiltration and RM “writers” (Additional file 1: Fig. S3B). For example, NSUN6, METTL14, and ZC3H13 were significantly negatively correlated with T Cells CD4 memory activated, while FTSJ1, METTL6, NSUN4 and WTAP were positively correlated with such cells. Then, differences in TME cell infiltration were calculated between the two RM clusters (Additional file 2: Table S6). As observed in Fig. 3H, the infiltration of macrophages M0 (*p* = 0.027) and T cells CD4 memory resting (*p* = 9.03 × 10^− 3^) was higher in Cluster_1. In line with this, M0 macrophage and T cell CD4 memory resting marker gene expression was considerably elevated in Cluster_1 (Fig. 3I). It was hypothesized that RM patterns impacted the degree of infiltration by various immune cell types.

### Construction of RM writer signature

**Fig. 4:**
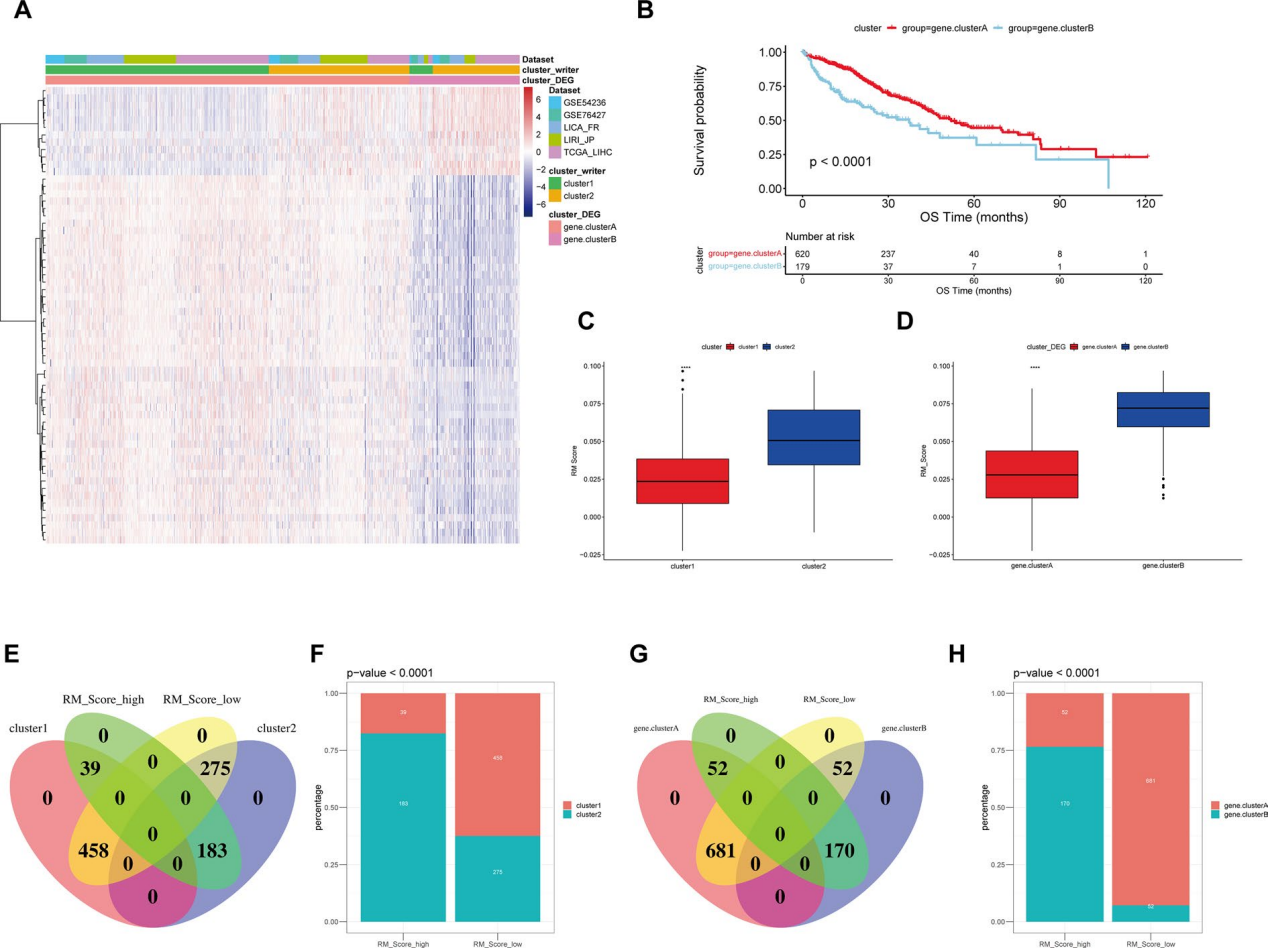
Construction of RM writers-related signature. **A** Unsupervised clustering of the RNA methylation-related genes. **B** Kaplan–Meier curves demonstrating the overall survival of gene.cluster_A and gene.cluster_B. **C** Comparison of RM_Score between Cluster_1 and Cluster_2. **D** Comparison of RM_Score between gene.cluster_A and gene.cluster_B. **E–H** The overlap and frequency of classifiers of RM_Score model with Cluster_1/2 and gene.cluster_A/B. ns, *p* > 0.05; *p < 0.05, **p < 0.01, ***p < 0.001, ****p < 0.0001

To further characterize the functional role of the two RM patterns above, we identified 62 RM phenotype-related DEGs and performed enrichment analysis. We discovered that these genes were enriched in metabolic activities, including drug catabolic process, alpha-amino acid metabolic process, and steroid metabolism (Additional file 1: Fig. S4A), which was also implicated in pentose phosphate pathway, glycolysis, PPAR signaling pathway (Additional file 1: Fig. S4B). The patients were then categorized into two genomic subtypes known as gene.cluster_A and gene.cluster_B using unsupervised clustering analyses based on the DEGs (Additional file 1: Fig. 4A; Additional file 2: Table S7). As shown in the survival analysis in Fig. 4B, patients at the subgroup of gene.cluster_ B had a worse prognosis than those in gene.cluster_A (*p* < 0.0001). We then developed an RM_Score algorithm to characterize the RNA modification profile of individual HCC patients based on these RM-related DEGs. We found that RM_Score of Cluster_2 and gene.cluster_B were significantly higher than Cluster_1 and gene.cluster_A (Fig. 4C, D). To further assess the clinical relevance of the RM_Score, we divided patients into RM_Score-low and -high groups with the cut-off value of 0.05859662 determined by the “surv_cutpoint” algorithm of “survminer” package (Additional file 2: Table S8). We performed overlap analysis of these three different classifiers based on the Wayne diagram and the histogram of frequency distribution (Additional file 2: Table S9). As shown in Fig. 4E-H, these three computational techniques of categorization had a high degree of coincidence.

### Clinical characteristics associated with RM_Score in HCC

**Fig. 5:**
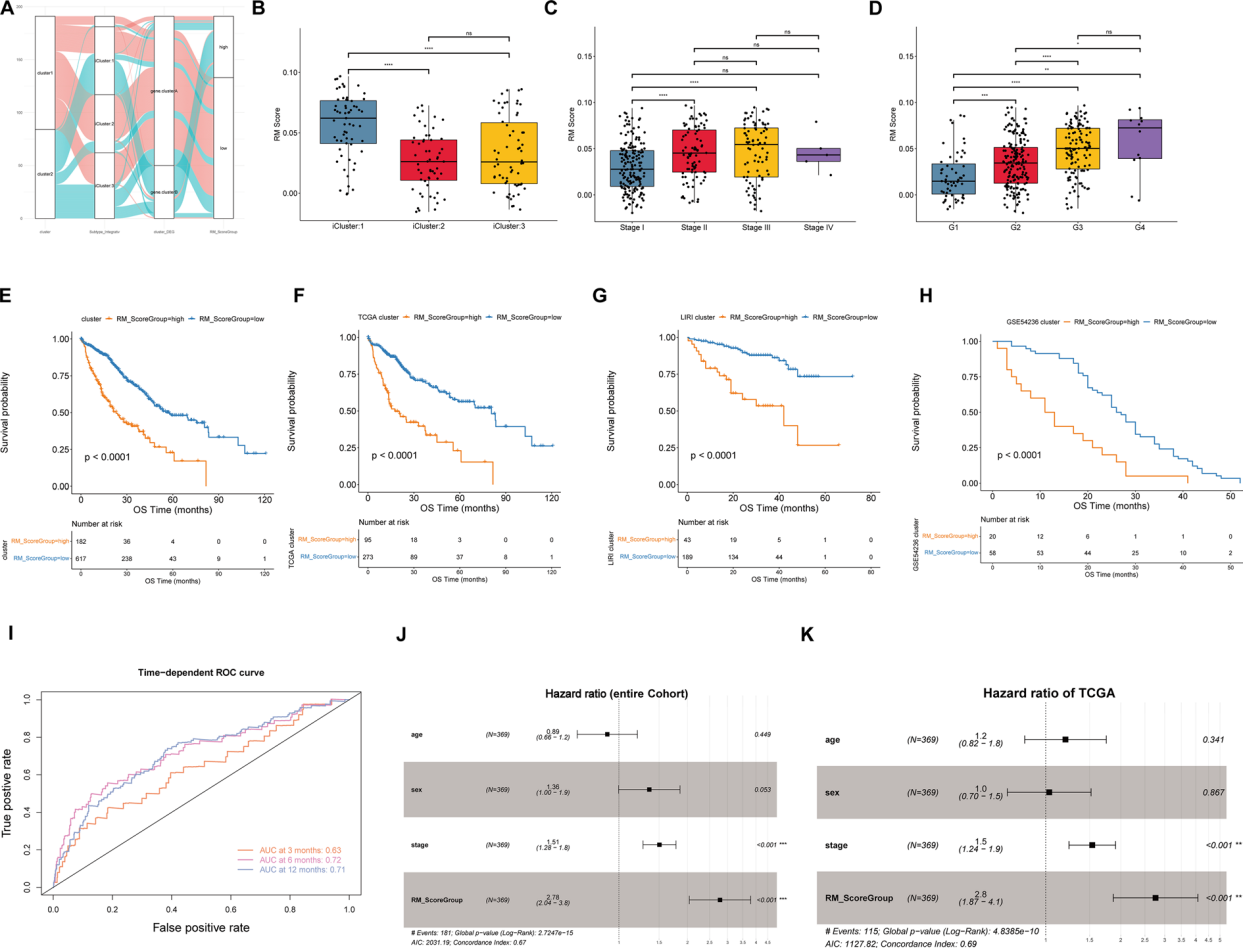
The clinical implications and prognostic value of the RM_Score system. **A** The correlation of molecular subtypes with Cluster_1/2, gene.cluster_A/B, and RM_Score high/low. **B-D** The comparison of RM_Score in patients at different iClusters, stages, and grades. **E** Kaplan–Meier curves demonstrating the overall survival of RM_Score high and RM_Score low subgroups in combined cohorts. **F–H** Kaplan–Meier curves demonstrating the overall survival of RM_Score high and RM_Score low subgroups in TCGA, LIRI, and GSE54236 cohorts, respectively. **I** The ROC curves of the RM_Score in predicting the overall survival of the liver cancer patients. **J**, **K** the Multivariate Cox regression model analysis to evaluated RM_Score as an independent prognostic factor in combined cohorts and TCGA-LIHC cohort. ns, *p* > 0.05; *p < 0.05, **p < 0.01, ***p < 0.001, ****p < 0.0001

HCC can be divided into 3 molecular subtypes, named iCluster1–3 with distinct molecular features. Alluvial diagrams were plotted to display the association between different classifiers and the subtypes (Fig. 5A, Additional file 2: Table S10). To examine the association between the RM_Score and subtypes, we then calculated the RM_Scores of different subtypes. As shown in Fig. 5B, RM_Scores dramatically varied among subtypes, with iCluster 1 achieving the highest score. Additionally, more advanced stages and grades of HCC cases were associated with higher RM_Score (Fig. 5C, D), implying potential relevance for RM patterns with tumor progression. As elucidated in Fig. 5E-H, patients with higher RM_Score experienced worse survival in the pooled cohorts or single cohort of the TCGA, ICGC, and GSE54236. At 3, 6, and 12 months of overall survival, the AUCs of ROC curves based on RM_Score were 0.63, 0.72, and 0.71, respectively (Fig. 5I). Then RM_Score was examined using multivariate Cox regression analysis as an independent prognostic factor. In the combined cohorts, RM_Score (Fig. 5J; HR = 2.78, 95% CI 2.04–3.8, *p* < 0.001), together with stage, were independent prognostic biomarkers. Consistently, RM_Score (Fig. 5K; HR = 2.8, 95% CI 1.87–4.1, *p* < 0.001) and stage were also defined as independent markers in TCGA cohort.

### Pathways and immune activity with RM_Score

**Fig. 6:**
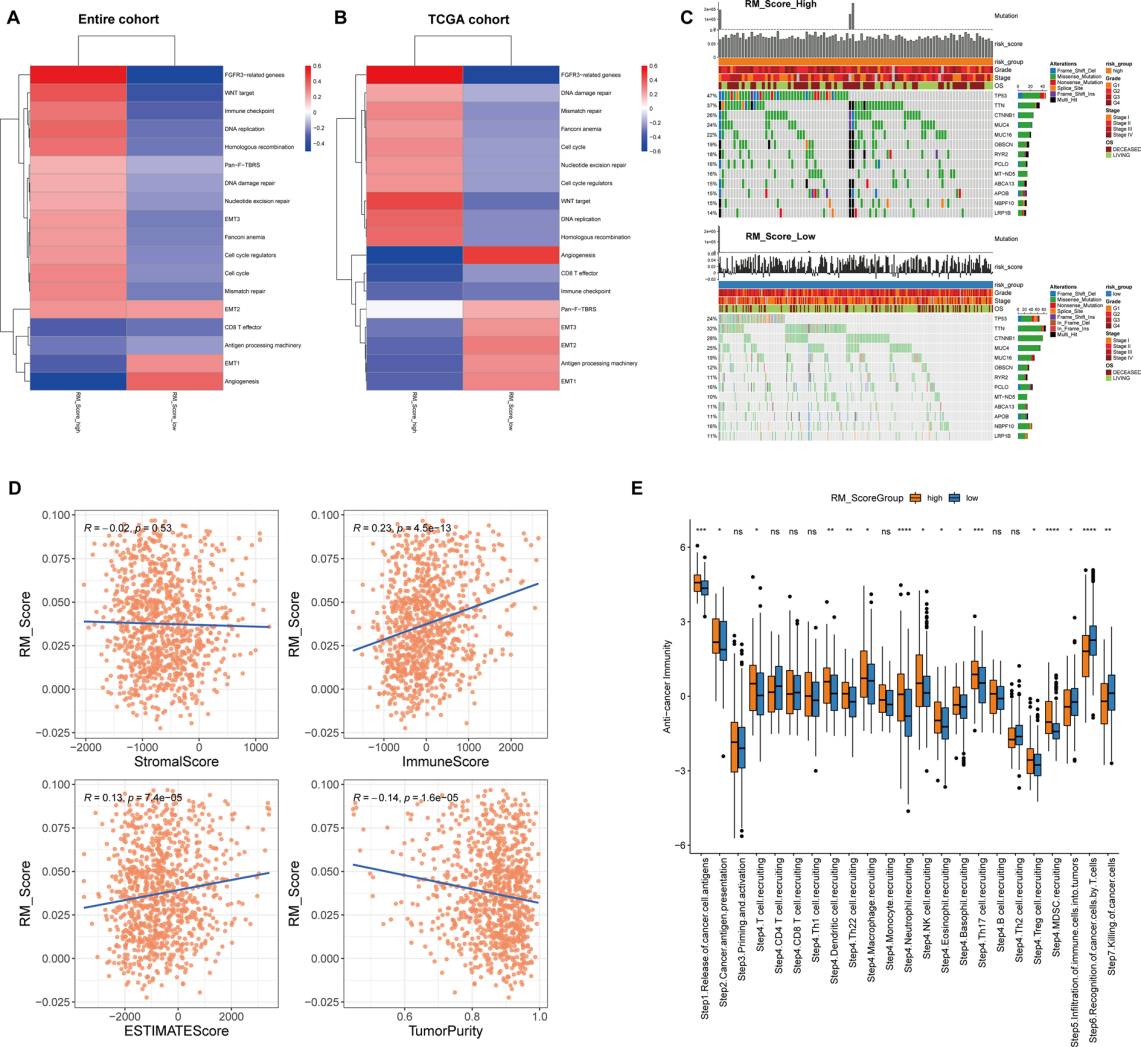
Potential characteristics underlying the RM_Score. **A**, **B** The differences of enriched signaling pathways between RM_Score-high and -low subgroups in combined datasets and the TCGA-LIHC dataset. **C** The mutational genes with top frequency in RM_Score high and low subgroups. **D** The correlation of RM_Score with STROMAL score, Immune score, Estimate score, and tumor purity. **E** the Tracking Tumor Immunophenotype analysis of RM_Score high and low subgroups

Then, we initially compared the two RM_Score subgroups-related pathways in the combined cohorts. DNA replication, cell cycle, immune checkpoints, DNA repair, the Wnt pathway, and DNA replication were enriched in the RM_Score-high group, whereas angiogenesis and antigen processing were involved in the RM_Score-low group (Fig. 6A). In TCGA cohort, the DEGs between RM_Score high and low groups were also predominantly enriched in proliferation- and immune-related pathways (Fig. 6B). Furthermore, we also explored into whether the two groups differed in terms of mutation. Although the two groups had identical altered genes, as illustrated in Fig. 6C, the mutation frequency and type significantly varied, particularly for TP53, TTN, CTNNB1, and MUC4/16. Additionally, we identified a connection between RM_Score and immune cells in TME. Neutrophil and activated CD4 T cell proportions showed the most notable differences in cell proportion (Additional file 1: Fig. S5). The immune score and estimate score were both positively correlated with RM_Score, whereas tumor purity was considerably adversely correlated with this score (Fig. 6D; Additional file 2: Table S11). Tracking Tumor Immunophenotype (TIP) discovered an obvious distinction between RM_Score high and low groups (Fig. 6E). The RM_Score-high group was triggered in Step1.Releasing of cancer cell antigens, Step4.Th22 cell.recruiting, Step4.Neutrophil.recruiting, Step4.Th17 cell.recruiting, and Step4.MDSC.recruiting. Contrarily, Step6.Recognition of cancer cells by T cells, Step7. Killing of cancer cells were implicated in RM_Score-low group.

### The RM_Score model predicts response to immunotherapy

**Fig. 7:**
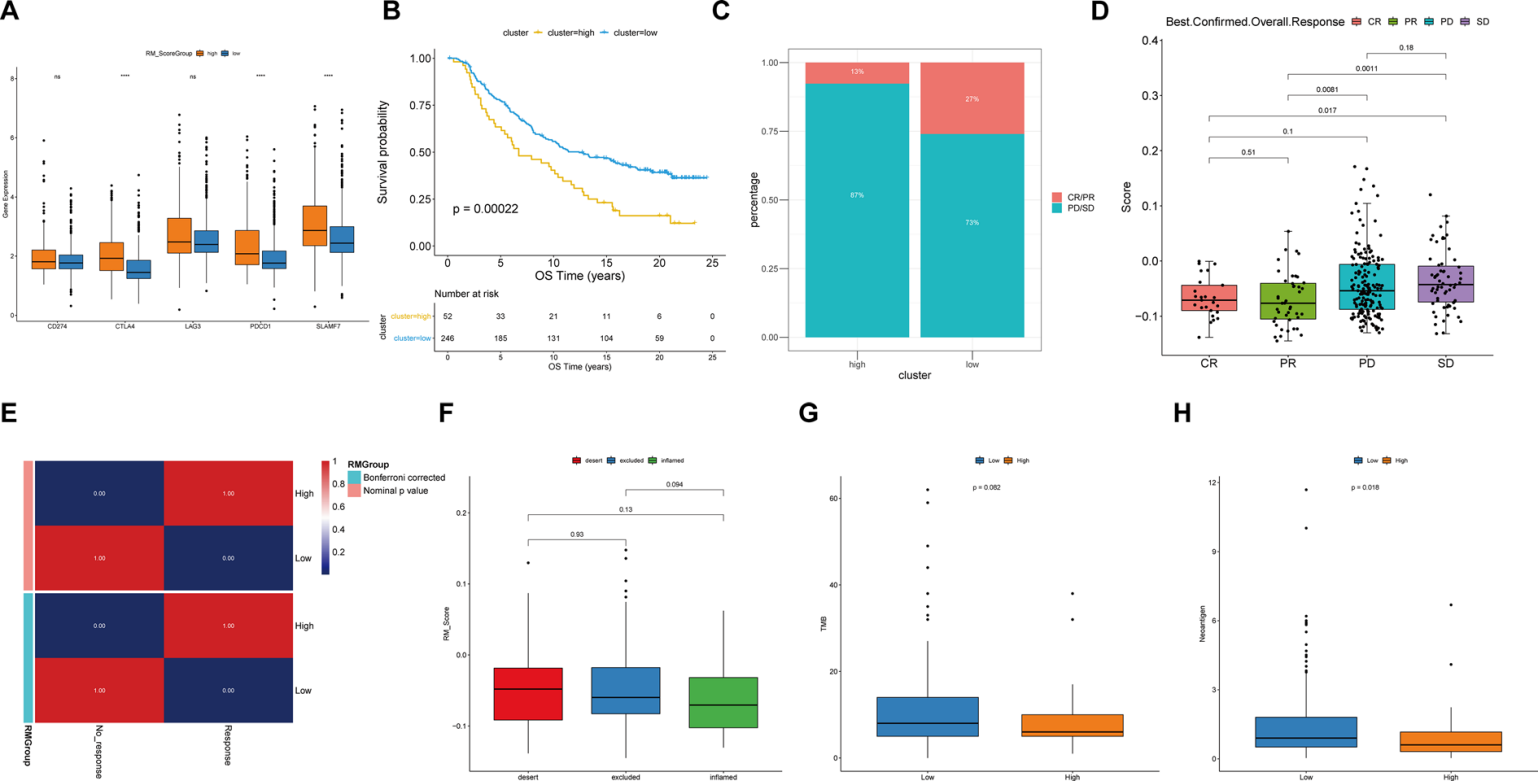
The capacity of RM_Score model in predicting immunotherapy response. **A** The expression of immune-related genes in RM_Score-high and -low subgroups. **B** Kaplan–Meier curves show overall survival in the RM_Score-high and -low subgroups after the PD-L1 immunotherapy in the IMvigor210 cohort. **C** The proportion of patients in the IMvigor210 cohort with different responses to PD-L1 blockade immunotherapy. **D** The difference of clinical outcomes with anti-PD-L1 treatment in the RM_Score high and low subgroups in the IMvigor210 cohort. **E** The similarity of gene expression profiles between RM patterns and melanoma patients treated with ICB (*n* = 47). **F** The RM_Score in three immune subtypes of IMvigor210, including “immune inflamed”, “immune excluded”, and “immune desert”. **G**, **H** The TMB and neoantigen burden in the RM_Score-low and RM_Score subgroups. ns, *p* > 0.05; *p < 0.05, **p < 0.01, ***p < 0.001, ****p < 0.0001

Developing biomarkers to forecast immunotherapy response has been a top priority. The expression of the checkpoint genes was then discovered in the two groups. CTLA4, PDCD1 and SLAMF7 were significantly elevated in high RM_Score group (Fig. 7A). Considering the potential association between RM_Score and immunological milieu, we examined the predictive capacity of the RM_Score towards ICB therapy. In the IMvigor210 cohort, we discovered that patients with low RM_Score (cut-off value = 0.002636961) showed substantially therapeutic benefits and had a noticeably extended overall survival (Fig. 7B, *p* = 0.00022). Additionally, the 348 individuals in the IMvigor210 cohort responded to anti-PD-L1 blockers with varying degrees (Additional file 2: Table S12). As shown in Fig. 7C, patients with high RM_Scores exhibited larger ratios of stable disease (SD) and progressive disease (PD) than patients with low RM_Scores. Additionally, the patients with complete response (CR) and partial response (PR) showed reduced RM_Score than SD and PD patients (Fig. 7D; Additional file 2: Table S13). Submap analysis indicated that the low RM_Score group was more inclined to respond to anti-PD-1 treatment (Fig. 7E). We further calculated the RM_Score of the three immune subtypes of IMvigor210, including “immune inflamed”, “immune excluded”, and “immune desert”. The results revealed that the “immune inflamed” type had lower RM_Score than two other groups (Fig. 7F). Likewise, the TMB and neoantigen burden were considerably higher in the group with a low RM_Score than in the group with a high RM_Score (Fig. 7G, H), indicating a potential correlation between the RM_Score model and immunotherapy.

### Potential therapeutic value of the RM_Score and ceRNA network development

**Fig. 8:**
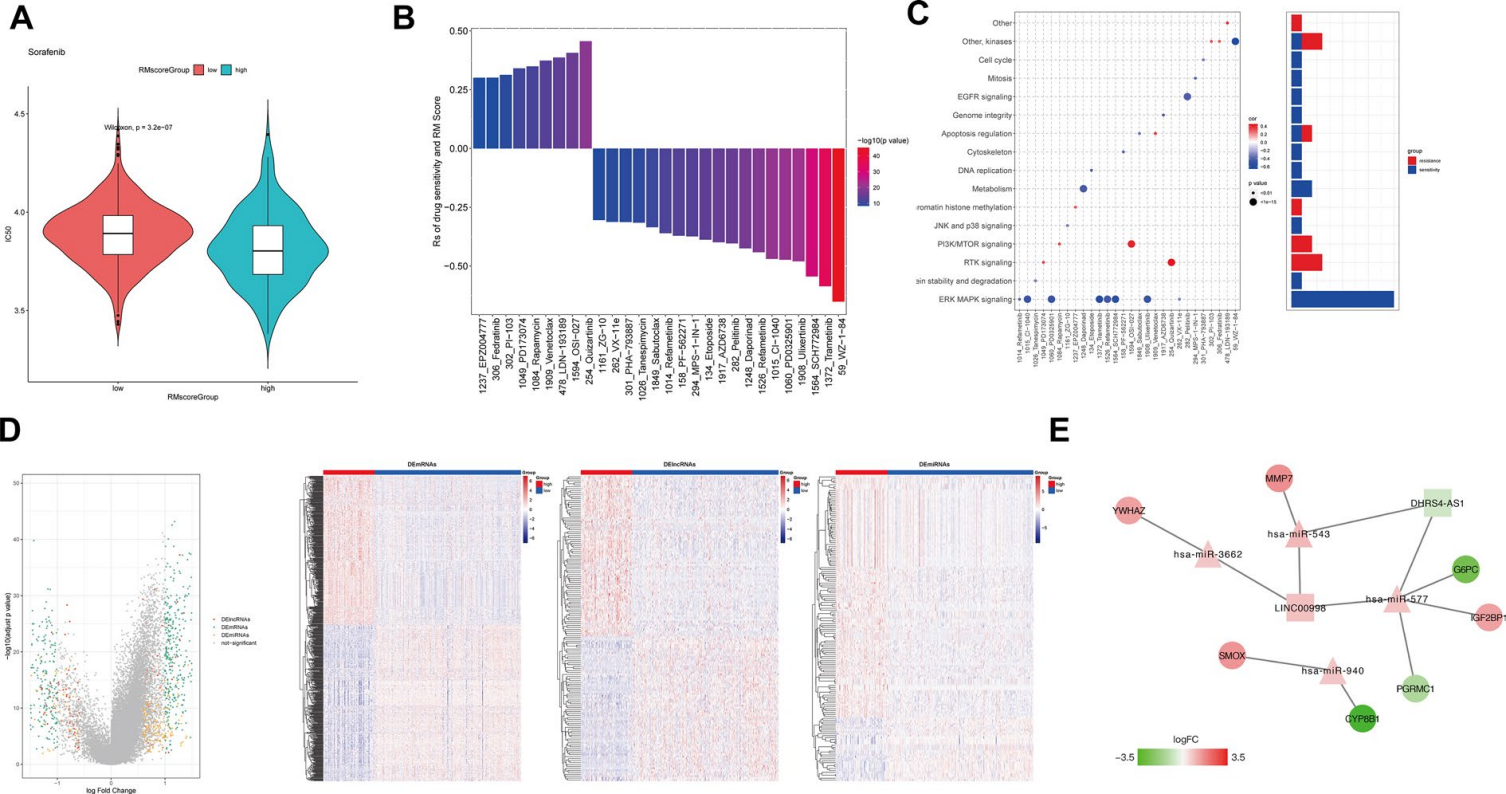
RM_Score-related Therapeutic sensitivity and ceRNA network. **A** IC50 values of sorafenib in RM_Score high and low groups in Genomics of Drug Sensitivity in Cancer (GDSC) cell line dataset. **B** The Spearman correlation analysis discovered the association between the RM_Score and drug sensitivity in the GDSC database. **C** The signaling pathways targeted by these RM_Score sensitive drugs. **D** The volcano map and heatmaps elucidating the differentially expressed mRNA, miRNA, lncRNA between RM_Score high cases and RM_Score low cases **E** The ceRNA network consisting of 2 DElncRNAs, 4 DEmiRNAs and 7 DEmRNAs

Given the availability of targeted therapy for HCC, we assessed sorafenib sensitivity in both groups. To ensure the stability of the prediction results, prediction models were trained using the Genomics of Drug Sensitivity in Cancer (GDSC) cell line dataset by ridge regression and validated using tenfold crossover. For each sample, subsequently, IC50 values were calculated and the discrepancies were compared. We identified that the high score group in the discovery cohort was more likely to be responsive to sorafenib (Fig. 8A, *p* = 3.2 × 10^−7^). In the GDSC database, we found 28 strongly associated pairs between RM_Score and drug sensitivity using the Spearman correlation analysis (Fig. 8B). Among them, 9 pairs showed that drug resistance correlated with the RM_Score, e.g., FLT3 inhibitor (quizartinib, Rs = 0.46, *p* = 2.28 × 10^−20^), BCL2 inhibitor (venetoclax, Rs = 0.37, *p* = 3.41 × 10^−13^), mTOR inhibitor (OSI-207, Rs = 0.41, *p* = 3.94 × 10^−16^; PI-103, Rs = 0.31, *p* = 8.0 × 10^−10^; rapamycin, Rs = 0.35, *p* = 4.86 × 10^−12^) and ALK inhibitor (LDN-193189. Rs = 0.39, *p* = 1.24 × 10^−14^). 19 pairs showed drug sensitivity, e.g., MEK inhibitor (trametinib, Rs =  − 0.59, *p* = 2.57 × 10^− 35^; refametinib, Rs =  − 0.44, *p* = 5.18 × 10^− 19^; PD0325901, Rs =  − 0.47, *p* = 5.70 × 10^− 22^; CI-1040, Rs =  − 0.47, *p* = 1.37 × 10^− 21^) and ERK inhibitor (SCH772984, Rs =  − 0.54, *p* = 7.44 × 10^− 30^; ulixertinib, Rs =  − 0.48, *p* = 1.26 × 10^− 22^). Further, we analyzed the signaling pathways of the genes targeted by these drugs. We discovered that compounds that were sensitive to high RM_Score primarily targeted the signaling pathways for MAPK/ERK and metabolism. In contrast, low RM_Score associated drugs targeted RKT and PI3K/mTOR signaling pathway (Fig. 8C). Thus, it indicated that RNA methylation patterns are correlated with drug sensitivity, which provides potential treatment strategies. In order to investigate the RM_Score-related potential key axis, we further constructed the ceRNA network based on the identification and validation of the clinical implications. By mincing the TCGA dataset, 735 differentially expressed mRNA (|logFC|> 1, *p* < 0.05), 165 differentially expressed miRNA (|logFC|> 1, *p* < 0.05) and 302 differentially expressed lncRNA (|logFC|> 1, *p* < 0.05) were identified between high RM_Score and low RM_Score samples (Fig. 8D). By utilizing Cytoscape, 2 DElncRNAs, 4 DEmiRNAs and 7 DEmRNAs were used to construct the ceRNA network (Fig. 8E, Additional file 2: Table S14).

## Discussion

Methylation modification is a common procedure that occurs in practically all biological processes. Methylation modifications on DNA and proteins have been thoroughly investigated during the past few decades [22, 23]. The characteristics and mechanisms of various RNA methylation modifications on eukaryotic RNA, however, are still largely unknown. The involvement of RNA methylation modifications dysregulation in several diseases, particularly malignancies, are being highlighted by a growing body of studies [24]. There have been several “writers” of RNA methylation modifications that have been associated with the onset or progression of liver cancer [25, 26]. A thorough study of the characteristics of RNA methylation modifications might enable to improve survival prediction and uncover potential molecular targets for HCC. In the current study, for the first time, we provided a comprehensive overview of five RNA methylation alteration types in HCC, with an emphasis on the writers. According to the mutation data of TCGA, 20 mutated writers were correlated with poor survival of HCC patients, of which KIAA1429 and FTSJD2 had highest mutation frequency. DEGs between HCC patients with and without writer mutation were mainly enriched in proliferation and inflammatory pathways. While two m^5^C writers, NSUN6 and TRDMT1, were downregulated in HCC tissues, the majority of writers were overexpressed in HCC samples. Subsequent CNV analysis revealed significant genetic heterogeneity and RNA modification "writer" expression discrepancies between the normal and HCC samples.

Following the exclusion of missing genes, 35 writers were recruited to investigate the expression pattern in five HCC datasets. Positive interactions were discovered among the authors, indicating a cross-talk of the various RNA methylation modification types. HCC individuals were categorized into two clusters based on consensus clustering, with distinct overall survival. Additionally, Cluster_1 was enriched in RNA processing pathways, whereas Cluster_2 was implicated in metabolic pathways. Next, we examined the probable functions of “writers” in TME. Diverse immune cell types were infiltrated into two clusters, indicating that RNA modification was involved in the infiltration of particular immune cell types.

Then, using unsupervised clustering, we established two genomic subgroups using RNA methylation modification-related DEGs. A poorer prognosis was attributed to gene.cluster B. Additionally, we generated an RNA methylation modification DEGs-based model to score HCC individuals in the pooled cohort, which was then divided into two groups based on the RM_Score. This stratification using the RM_Score was particularly related to the other two computational methods. The relationship between the RM_Score and clinical parameters was then assessed. Molecular subtype iCluster 1 had a higher RM_Score than iCluster 2/3. According to a previous study, iCluster 1 had a higher tumor grade, more macrovascular invasions, and a considerably worse prognosis than the other two clusters [27]. RM_Score is consistently elevated in HCC cases compared with higher grades or stages. Moreover, ROC and multivariate Cox regression analysis showed that the RM_Score model had remarkable prediction performance for the survival of HCC patients. In addition, patients with high RM_Score have significantly shorter overall in pooled or either dataset. It was hypothesized that the high RM_Score might be related to the clinical prognosis and HCC features. P53, widely acknowledged as a tumor suppressor, is inactivated with mutation, which promotes tumor progression [28]. It is interesting to note that the prevalence of TP53 mutations in the RM_Score-high group was more than twice as high as that in the RM_Score-low group, pointing to a possible link between aggressive traits related to RM_Score and TP53 mutation.

On the basis of the enrichment analysis, we further investigated the relationship between RM_Score and tumor immunity. Although there was no discernible difference in the proportion of immune cells between the groups, RM_Score was favorably associated with immune score, estimate score, and tumor immunophenotypes including Th22/Th17 cell recruitment, release of cancer cell antigens, and neutrophil/MDSC recruitment. It also implied a connection between RM_Score and tumor immune. Nowadays, immunotherapy is gaining popularity as a promising treatment option for advanced HCC with immune escape [29, 30]. In light of this, we assessed the RM_Score model's accuracy in predicting immunotherapy response. Patients with low RM_Scores may benefit from ICB treatment and have significantly longer overall survival. Patients with high RM_Score, on the other hand, showed less response after therapy, indicating a correlation between RM_Score and ICB resistance. In addition, RM_Score was varied in different levels of TMB and neoantigen burden, which were frequently correlated with immunotherapy efficacy. Thus, the RM_Score is a candidate model for predicting ICB response.

Tyrosine kinase inhibitor (TKI) sorafenib was approved by the USA FDA in 2007 for the treatment of advanced HCC. Increasing oral multi-targeted TKIs were subsequently awarded permission for use in treating HCC worldwide [31, 32]. Furthermore, we evaluated the relationship between RM_Score and Sorafenib sensitivity. Interestingly, the group with a high RM_Score demonstrated enhanced sorafenib sensitivity. The administration of sorafenib might be advantageous for those who experienced ICB resistance. For other targeted drugs, according to the analyses in GDSC database, high RM_Score drug indicated resistance to BCL2 inhibitor, JAK inhibitor, mTOR inhibitor and ALK inhibitor, while low RM_Score showed drug sensitivity of MEK inhibitor and ERK inhibitor. Thus, it indicated that RNA methylation patterns are correlated with sensitivity of targeted drug, providing potential treatment strategies for HCC patients. Additionally, a ceRNA network associated with RM_Score was developed to further explore clinical implications or malignant behaviors. It should be noted that LINC00998 and DHRS4-AS1 were found to be hub lncRNAs that were associated with miRNAs and mRNAs. Multiple tumor types have been found to be suppressed by LINC00998 [33, 34]. However, LINC00998-encoded peptide accelerated HCC tumorigenesis and facilitated aggressive behaviors [35]. DHRS4-AS1 was also defined as an anti-oncogenic ncRNA, which could inhibit proliferation of HCC cells [36].

As previously stated, the findings presented here demonstrate that these RM networks enable connections between clinical and RNA modification by stratifying patients' prognosis and therapy responses. However, this study had some limitations as well. Though the promising conclusions were based on the integrative bioinformatics in multiple levels, future functional and mechanistic research on these RNA writers will reveal clinical phenotypes that are caused by RNA methylation writers. To fully illustrate the network of RNA methylation modifications, future studies should also include “readers” and “erasers” and other RNA methylation like m^7^G and A-I modification. In addition, larger and multi-center clinical cohorts should be employed to assess the accuracy of RNA methylation-based classifier and the RM_Score prognostic model.

## Conclusions

In conclusion, we have demonstrated that RNA methylation modification patterns may serve crucial role in the progression of liver malignancy and immune dysfunction. Additionally, the subgroup classification based on RNA methylation writers may distinguish individuals with poor survival and forecast the efficacy of immunotherapy or targeted therapy. Moreover, RNA methylation modification regulators are also highlighted as robust biomarkers or potential targets for HCC.

## Supplementary Information

**Additional file 1: Figure S1**. The survival status in patients with or without RM writer mutation. **A**, **B** The OS and DFS in HCC patients at mutation type and non-mutation type in TCGA-LIHC cohort. **C1-C38**, The OS in HCC patients with or without RM writers mutations in TCGA-LIHC cohort. **Figure S2.** The distribution of correlation coefficient between writer expression and CNV in HCC. The mRNA expression of the 38 RM writers in Normal, CNV_loss, None_CNV, and CNV_gain groups. **Figure S3.** The prognostic analysis of RM writers and correlation with TME cells. **A **The Univariate cox analysis to evaluate the correlation of RM writers with overall survival of HCC patients. **B** Heatmap showed the positive (red) and the negative (blue) correlation between TME infiltration and RM writers in HCC. **Figure S4.** The biological functions and pathways underlying the RM phenotype-related DEGs. **A** GO enrichment of the 62 RM phenotype-related DEGs. **B **KEGG enrichment analysis of the 62 RM phenotype-related DEGs. **Figure S5.** The correlation of RM_Score with TME infiltration. Heatmap shows the differences in TME infiltration between RM_Score-high and -low groups in the combined cohorts.

**Additional file 2: Table S1.** Information of HCC cohorts. **Table S2.** RNA Modification Writers. **Table S3.** Samples clustering in 5 HCC cohorts. **Table S4.** GSVA_KEGG analysis of the DEGs between the Cluster 1/2. **Table S5.** GSVA_HALLMARK analysis of the DEGs between the Cluster1/2. **Table S6.**  Difference of TME infiltration characteristics between Cluster_1 /2. **Table S7.** Gene clustering in the pooled HCC cohorts. **Table S8.** RM_ScoreGroup stratification of the pooled cohort. **Table S9.** Sample information of the three computational methods of classification in 5 HCC cohorts. **Table S10.** Association of subtype with three computational methods of classification in TCGA LIHC cohorts. **Table S11.** The immune-related score of the samples in 5 HCC cohort. **Table S12.** Clinical information of IMvigor210 cohort. **Table S13.** The RM_Score of the samples in IMvigor210 cohort. **Table S14.** The RM_Score related ceRNA network.

## Data Availability

All data generated or analyzed during this study are included in this published article.

## Abbreviations

HCC: Hepatocellular carcinoma
HBV: Hepatitis B virus
HCV: Hepatitis C virus
TCGA-LIHC: The Cancer Genome Atlas Liver Hepatocellular Carcinoma
NCBI: National Center for Biotechnology Information
GEO: Gene Expression Omnibus
RM: RNA methylation
PTM: Post-translational modification
m^1^A: N1-Methyladenosine
m^5^C: 5-Methylcytosine
m^3^C: N3-Methylcytidine
m^6^A: N6-Methyladenosine
Nm: 2′-O-Methylation
DEGs: Differentially expressed genes
ceRNA: Competing endogenous RNA
GSVA: Gene set variation analysis
CNV: Copy number variation
GDSC: Genomics of Drug Sensitivity in Cancer
