# Supplementary material for: Integrated investigation of the clinical implications and targeted landscape for RNA methylation modifications in hepatocellular carcinoma
Source: Eur J Med Res. 2023 Jan 27;28:46. doi: 10.1186/s40001-023-01016-7 (PMC9881284; doi:10.1186/s40001-023-01016-7)
Supplement: Supplementary file 1 — Additional file 1: Figure S1. The survival status in patients with or without RM writer mutation. A, B The OS and DFS in HCC patients at mutation type and non-mutation type in TCGA-LIHC cohort. C1-C38, The OS in HCC patients with or without RM writers mutations in TCGA-LIHC cohort. Figure S2. The distribution of correlation coefficient between writer expression and CNV in HCC. The mRNA expression of the 38 RM writers in Normal, CNV_loss, None_CNV, and CNV_gain groups. Figure S3. The prognostic analysis of RM writers and correlation with TME cells. A The Univariate cox analysis to evaluate the correlation of RM writers with overall survival of HCC patients. B Heatmap showed the positive (red) and the negative (blue) correlation between TME infiltration and RM writers in HCC. Figure S4. The biological functions and pathways underlying the RM phenotype-related DEGs. A GO enrichment of the 62 RM phenotype-related DEGs. B KEGG enrichment analysis of the 62 RM phenotype-related DEGs. Figure S5. The correlation of RM_Score with TME infiltration. Heatmap shows the differences in TME infiltration between RM_Score-high and -low groups in the combined cohorts. [file 40001_2023_1016_MOESM1_ESM.docx]

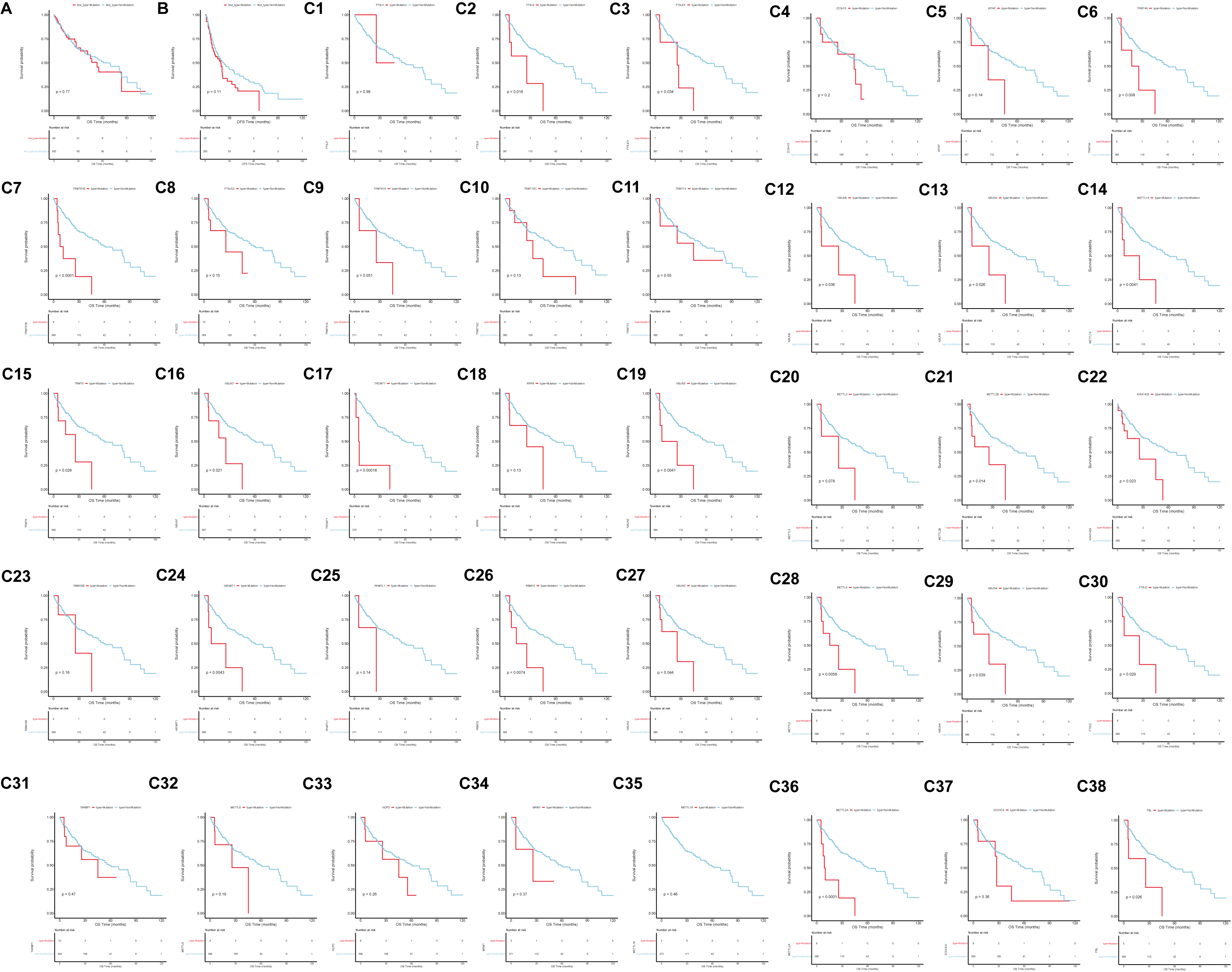


**Figure S1. The survival status in patients with or without RM writer mutation.**

**A&B,** the OS and DFS in HCC patients at mutation type and non-mutation type in TCGA-LIHC cohort.**C1-C38,** The OS in HCC patients with or without RM writers mutations in TCGA-LIHC cohort.


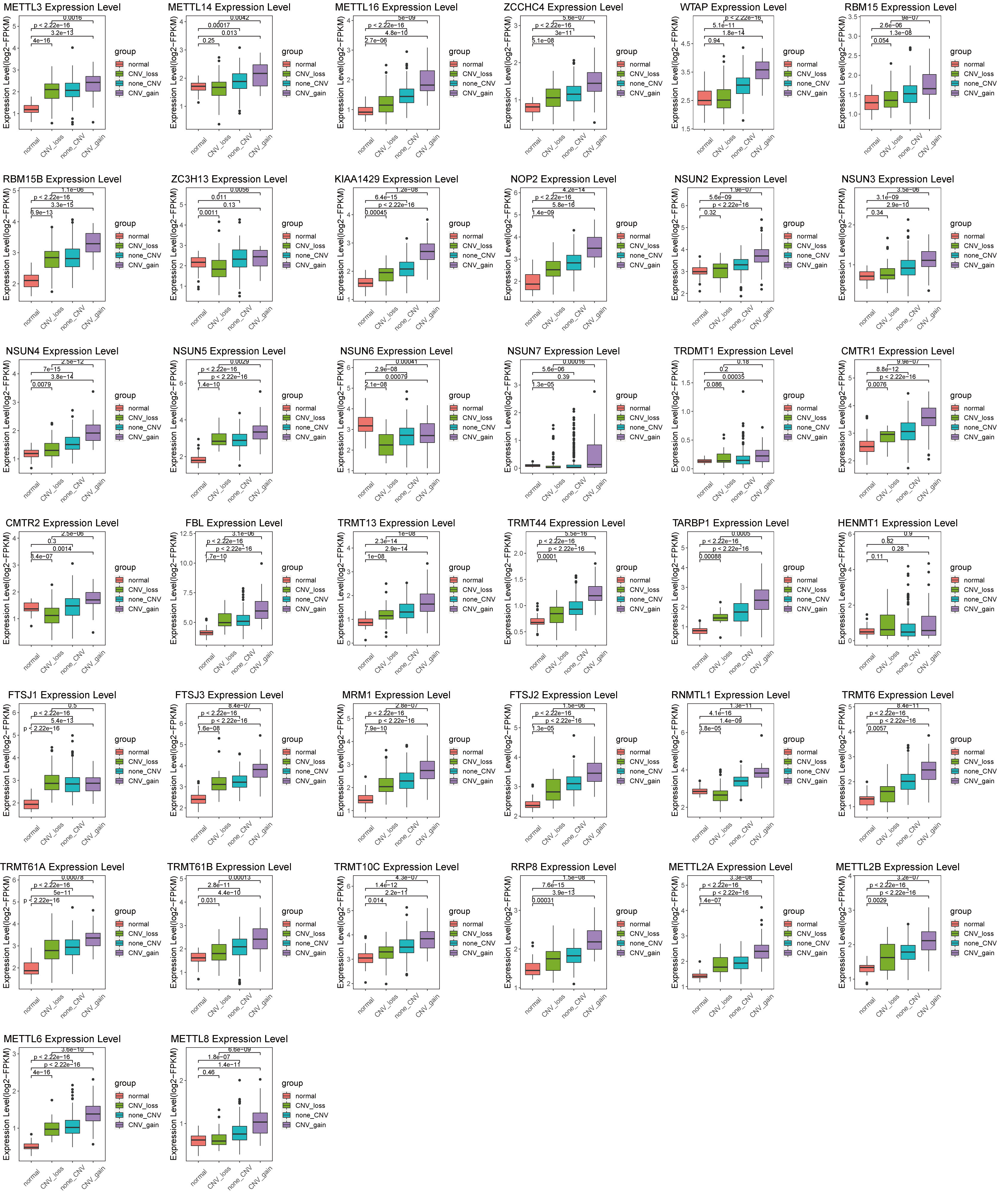


**Figure S2. The distribution of correlation coefficient between writer expression and CNV in HCC.** The mRNA expression of the 38 RM writers in Normal, CNV_loss, None_CNV, and CNV_gain groups.


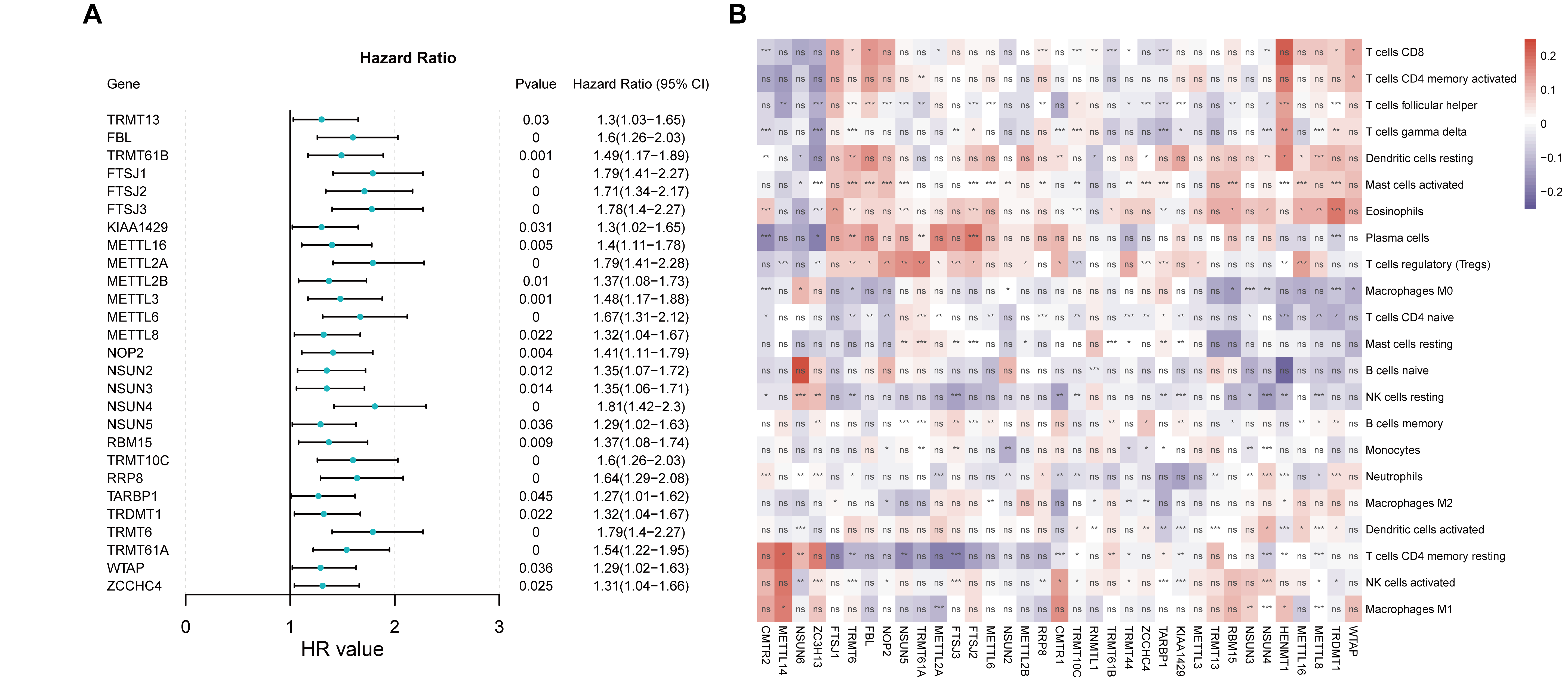


**Figure S3. The prognostic analysis of RM writers and correlation with TME cells.**

**A,** the Univariate cox analysis to evaluate the correlation of RM writers with overall survival of HCC patients. **B,** Heatmap showed the positive (red) and the negative (blue) correlation between TME infiltration and RM writers in HCC.


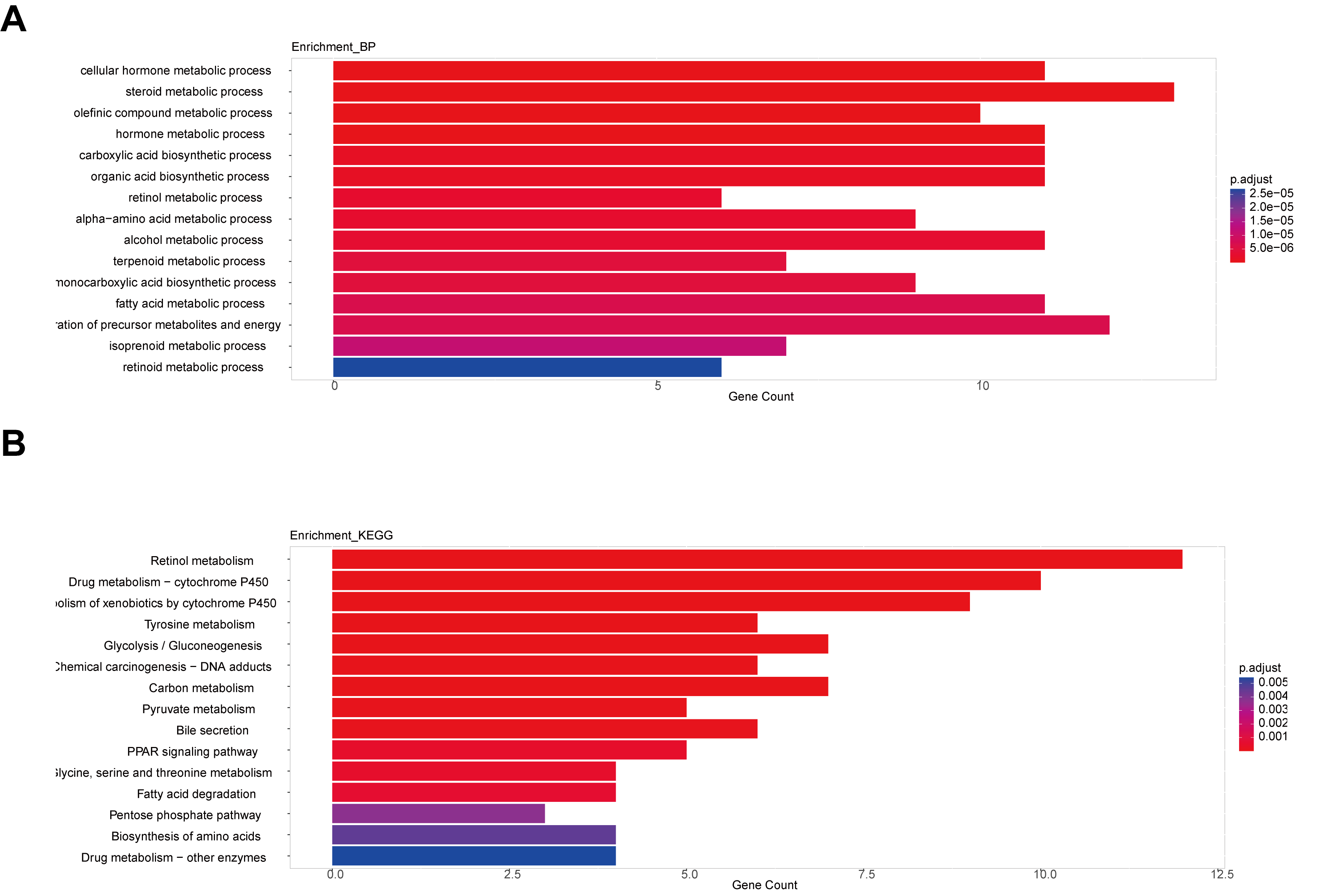


**Figure S4. The biological functions and pathways underlying the RM phenotype-related DEGs. A,** GO enrichment of the 62 RM phenotype-related DEGs. **B,** KEGG enrichment analysis of the 62 RM phenotype-related DEGs.


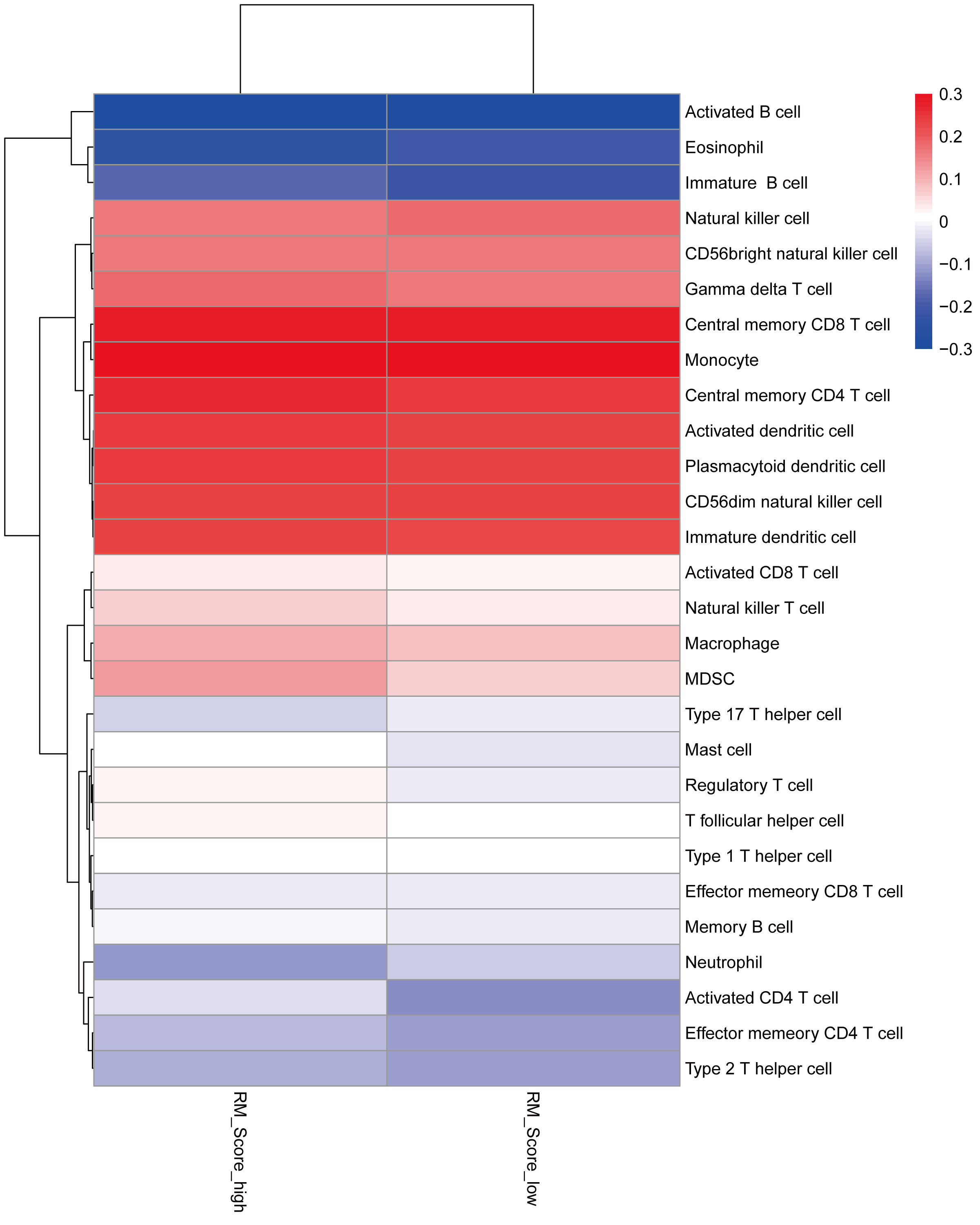


**Figure S5. The correlation of RM_Score with TME infiltration**. Heatmap shows the differences in TME infiltration between RM_Score-high and -low groups in the combined cohorts.
